# Maintaining robust size across environmental conditions through plastic brain growth dynamics

**DOI:** 10.1098/rsob.220037

**Published:** 2022-09-14

**Authors:** Ansa E. Cobham, Brent Neumann, Christen K. Mirth

**Affiliations:** ^1^ School of Biological Sciences, Monash University, Melbourne, Australia; ^2^ Neuroscience Program, Monash Biomedicine Discovery Institute and Department of Anatomy and Developmental Biology, Monash University, Melbourne, Australia

**Keywords:** whole brain, mushroom bodies, insulin signalling, growth rate, plasticity, robustness

## Abstract

Organ growth is tightly regulated across environmental conditions to generate an appropriate final size. While the size of some organs is free to vary, others need to maintain constant size to function properly. This poses a unique problem: how is robust final size achieved when environmental conditions alter key processes that regulate organ size throughout the body, such as growth rate and growth duration? While we know that brain growth is ‘spared’ from the effects of the environment from humans to fruit flies, we do not understand how this process alters growth dynamics across brain compartments. Here, we explore how this robustness in brain size is achieved by examining differences in growth patterns between the larval body, the brain and a brain compartment—the mushroom bodies—in *Drosophila melanogaster* across both thermal and nutritional conditions. We identify key differences in patterns of growth between the whole brain and mushroom bodies that are likely to underlie robustness of final organ shape. Further, we show that these differences produce distinct brain shapes across environments.

## Graphical abstract

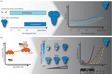

## 1. Introduction

How are the shapes and sizes of growing organs regulated throughout development to generate a fully functional multicellular animal with highly specialized parts? This seems particularly difficult to understand given that body parts initiate growth at different times, and further grow at different rates and with differing dynamics [1–3]. While some organs show exquisite sensitivity to environmental conditions, altering their shape and size with changes in nutrition, temperature, and other conditions, known as plasticity; other organs resist perturbations in environmental conditions and maintain relatively constant final sizes across conditions [4,5], a property that contributes to robustness in development [4–6]. As organs vary in sensitivity to environmental perturbations, animals that develop in different environments will differ in their body size and shape [7].

Extensive studies in insects have described how the patterns of growth across organs generate variation in size and shape of the adult body [1,6–8]. Growth dynamics can vary either at the level of an individual organ or through coordinating growth processes among various organs relative to the growing body [3,9,10]. Nutritional and temperature conditions from the environment also act to affect organ size [11–15].

Across a wide variety of animals, from insects to humans, the nervous system is generally less sensitive to changes in environmental conditions than other organs of the body [16–18]. This is commonly referred to as brain sparing [16]. Because of its short generation time and small size, the central nervous system of the fruit fly *Drosophila melanogaster*, hereafter referred to as the brain, has proven to be an excellent system to study the mechanisms of brain sparing.

**Figure 1.**
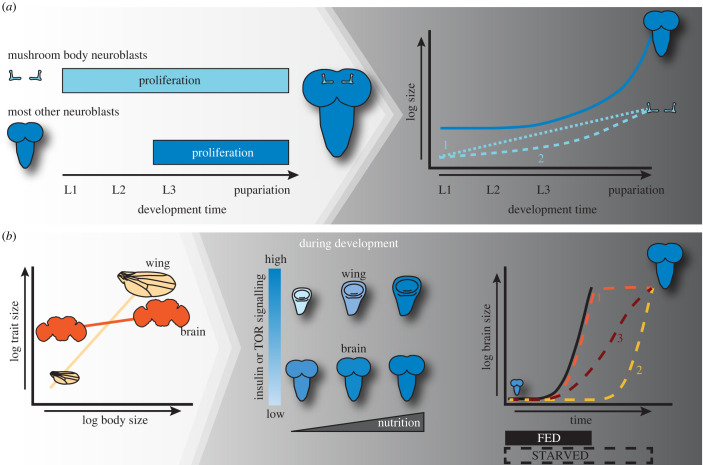
How do the growth dynamics of the whole brain and the mushroom bodies vary. (*a*) Hypothesis 1: Mushroom bodies proliferate throughout larval development, while most of the other neuroblasts in the brain remain quiescent and reinitiate proliferation in the late second instar (L2). These differences in proliferation could result in differences in the dynamics of mushroom body growth when compare to the whole brain. While we expect that the whole brain would show a lag period where it does not growth, followed by a period of exponential growth, the mushroom body might show constant (linear) increases in size across the larval stages of development (dashed line 1). Alternatively, the mushroom body might show similar growth dynamics, with shallower increase in growth rate in later development (dashed line 2). Differences in growth dynamics between the mushroom bodies and the whole brain would suggest that they are regulated in distinct manners under changing environmental conditions. (*b*) In comparison to other organs like the wing, adult brain size changes little with changes in body size. The reason that this is thought to occur is that insulin and TOR signalling is kept high in the brain even under poor-nutrient conditions via the action of Jeb/Alk. High levels of insulin or TOR signalling would suggest that brains would maintain constant growth rates even across environmental conditions—like starvation—that induce prolonged larval development. To maintain constant size, this would mean that the brain would either need to grow at constant rates until it reached its target size and then stop (orange dashed line 1), or else delay the onset of growth until later (yellow dashed line 2). Alternatively, Jeb/Alk could tune insulin or TOR signalling levels such that the rate of growth was reduced to compensate for the extended development time (red dashed line 3).

**Figure 2.**
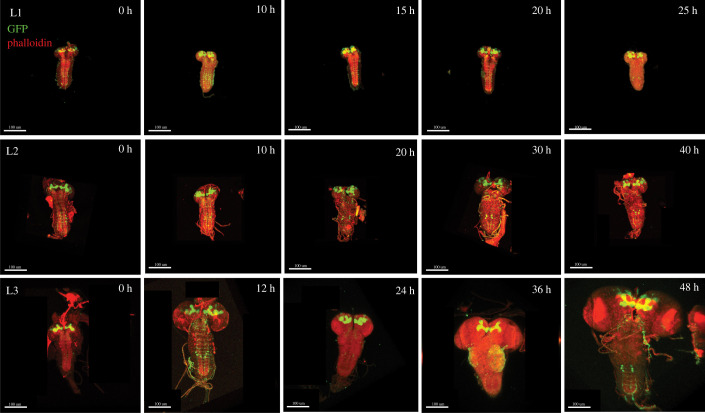
Changes in brain growth across larval stage of development. Larval brains expressing GFP in the neurons (green) of the mushroom body co-stained with phalloidin (red) across five developmental time points in the three larval stages. First instar (L1) (*a–e*) (0 h is relative to hatching), the second instar (L2) (*f–j*) (0 h relative to the moult to L2) and the third instar (L3) (*k–p*) (0 h relative to the moult to L3). At L3, the last two time points correspond to wandering and white prepupal stages. (Scale bar: 100 µm).

**Figure 3.**
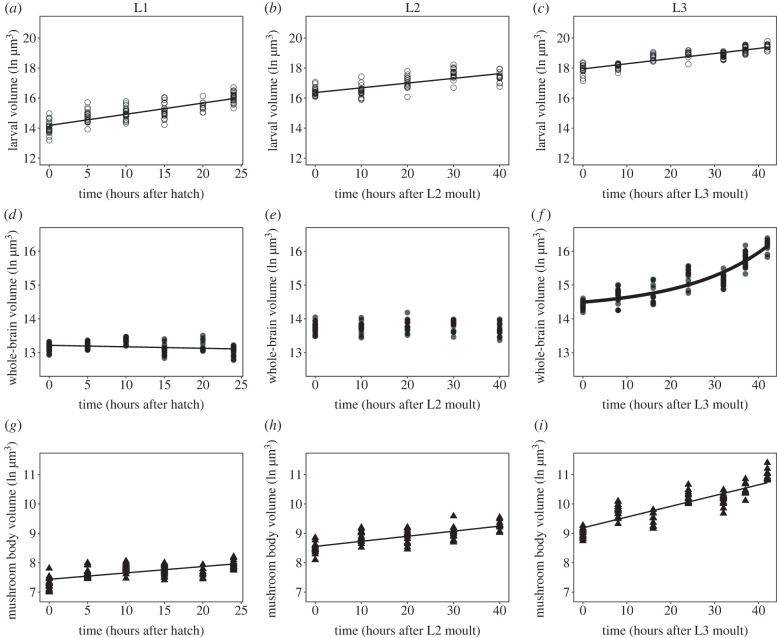
Growth patterns of larval body, brain and mushroom body. The volume of the larval body (*a–c*), whole brain (*d–f*) and mushroom body (*g–i*) at L1 stage (*a,d,g*), L2 stage (*b,e,h*) and at L3 stage (*c,f,i*) measured from 0 h after hatching/ larval moult to the end of the larval instar. At L3, the last two timepoints correspond to wandering and white pre-pupae larval stages. Each point shows individuals measured.

In *Drosophila,* the cells of the brain differentiate in the embryo. Reactivation of quiescent neuroblast and glial stem cells occurs early in the first larval stage of development and requires cell-autonomous nutrient signals [19]. After reactivation, most brain growth occurs in the three larval stages. While we know that the size of the brain is maintained even when larvae are starved, how brain grow rates change in response to changes in the duration of growth is unknown (figure 1*a*). figures 2 and 3.

Brains could achieve growth sparing by modifying its growth rates in several ways. The brain could maintain constant growth rates, but then stop growing once a target size is reached (figure 1*a*), resulting in a growth trajectory that reaches an asymptote when developmental time is extended due to environmental conditions. Alternatively, the time at which brain growth is initiated could be delayed when environmental conditions extend developmental time, but once initiated, the brain maintains constant growth rates (figure 1*a*). In this scenario, larval brains would show exponential growth trajectories across rearing conditions, where the time at which exponential growth is initiated depends on environmental conditions (lagged exponential model). Finally, the brain might tune its growth rates with the timing at which growth is initiated to adjust for extended growth period caused by environmental conditions (figure 1*a*). This would result in change in both growth rates and the time at which growth was initiated.

Like all brains, the *Drosophila* larval brain is composed of distinct functional compartments, each containing specific populations of neurons [20,21], that differ in their growth properties [22] and vary their rates of cell division in response to nutrition and other environmental conditions like temperature, light and population densities during larval stages of growth [23–26]. Specific neuronal subclasses act to sense amino acids in the environment and regulate the onset of pupariation during the period of nutrient restriction [27,28]. As most neuroblast populations enter quiescence in the early larval stages, the neuroblasts that give rise to the mushroom bodies—the paired neuronal structures important for olfactory processing and learning—continue to divide and differentiate from the first instar (L1) stage onwards [29]. Even when larvae are starved, the mushroom body neurons continue to divide, but do not differentiate [24,30]. In contrast with the mushroom bodies, the optic lobe neurons, that receive sensory input from the visual system, are only activated late in larval development and their cell division patterns are highly sensitive to changes in the nutritional environment [31]. This suggests that specific brain regions differ in how they protect the whole brain from environmental perturbations.

We can therefore propose a model of how the mushroom body compartments of the brain might maintain constant size in the face of changing environmental conditions. First, mushroom body neuroblasts begin proliferating much earlier than majority of other brain neuroblasts, throughout the larval instars, so we expect the size of these structures to increase constantly, or linearly, throughout larval development (figure 1*b*). As the remaining neuroblasts of the brain initiate proliferation late in the second instar, we would an exponential growth with a time lag to its onset in the whole brain, where a period of little or no discernible brain growth in the first two instars is punctuated by a rapid onset of growth in later development (figure 1*b*). This would contrast with the patterns of growth predicted to occur throughout the whole brain (figure 1*b*) and would mean that growth dynamics could be mediated by differing mechanisms across different compartments.

In the current study, we aim to establish how brain growth dynamics are regulated, to ensure robust final brain size across different environmental conditions, and whether all compartments of the brain are regulated in the same manner. To address this, we compared the growth patterns of whole brains and mushroom bodies, relative to the larval body, under standard rearing conditions. We then used altered nutritional and thermal conditions to explore how growth dynamics respond to environmental change. These studies reveal differences in the way the mushroom body compartment regulates its growth when compared to the whole brain and highlight how growth dynamics are tuned by nutrition and temperature. Further deepening our understanding of how different brain regions maintain robustness across environmental conditions.

## 2. Results

### 2.1. Comparing the growth dynamics of the larval body, whole brain and mushroom bodies across larval development

Given that the mushroom body neuroblasts initiate growth and respond to nutrition differently than the majority of other neuroblasts in the brain, our first goal was to devise methods to compare mushroom body growth to whole brain and larval body growth across all three larval instars. To ensure that we compared the growth of the same structures across developmental time, we required a marker for mushroom body neurons that would be expressed throughout all three instars. Using the expression data available from the Janelia FlyLight project (http://flweb.janelia.org/cgi-bin/flew.cgi), we found that the GMR38E10 GAL4 line drove green fluorescent protein (GFP) expression in the vertical and medial lobes of the mushroom body neurons from hatch through to pupariation (electronic supplementary material, figure S1). In the late L3 stage, GFP expression was not apparent in the mushroom body calyx (electronic supplementary material, figure S1), which is the dendritic projections of Kenyon cell bodies (electronic supplementary material, figures S2a and S2b). Thus, to be able to compare measurements across all stages of development, we excluded the calyx and peduncles from our analyses and measured only the ventral and medial lobes for mushroom body volume (electronic supplementary material, figure S2a and S2b).

We next sought to compare the dynamics of larval, whole brain and mushroom body growth. Log-transformed larval growth increased steadily throughout the first-, second- and third-instar stages (figure 3*a*–*c*; electronic supplementary material, table S1). Linear models explain 68%, 55% and 78% of the variation in larval volume over time for L1, L2 and L3, respectively (electronic supplementary material, table S1, adjusted *R*^2^ values). Similarly, the mushroom body displayed steady linear growth throughout all three instars (figures 2 and 3*g*–*i*; electronic supplementary material, table S1), with linear models explaining 43%, 55% and 77% of the variance in mushroom body volume over time for the L1, L2 and L3, respectively (electronic supplementary material, table S1, adjusted *R*^2^ values). By contrast, for whole brain volume, we observed a slight, but significant, decrease in whole brain volume with time in the L1 (figures 2 and 3*d*; electronic supplementary material, table S1). In this case, the linear model explained only 4% of the variance in whole brain volume in the L1 (electronic supplementary material, table S1, adjusted *R*^2^ values). There was no significant change in brain volume with time across the L2 stage (figure 3*e*; electronic supplementary material, table S1). In the L3, whole brain volume shows a nonlinear relationship with time, curving upwards. This suggests that whole brain growth speeds up as the third-instar progresses (figure 3*f*). Curiously, at 0 h after the moult to both L2 and L3, brain volume appears to increase despite no evidence of positive growth during the L1 or L2 instars. We cannot tell whether this is a random sampling effect or if this results from a burst of growth during the moult cycle itself, which we could not accurately sample.

Our results thus far suggest that whole brain growth is regulated differently to that of the larval body and mushroom bodies. To formally test this, we fit our growth data with both linear models and a range of nonlinear models commonly used to describe growth dynamics, including second-order polynomial, exponential, lagged exponential and power models [32]. Each of these models infers something different about growth. The second-order polynomial model assumes that the organ will have periods where its growth increases steadily with time, as well as periods during which growth rates slow down; exponential models describe growth that speeds up exponentially over time; lagged exponential models are similar to exponential models, but infer a period of slow or no growth followed by a switch to exponential growth; and the power model implies that growth increases according to a power function. We assessed which model best fit our growth data for each trait using two different model selection methods: the Akaike information criterion (AIC) and the Bayesian information criterion (BIC), both of which estimate the quality of each model relative to the others, penalizing models with a higher number of parameters to avoid overfitting the data. The model with the lowest AIC and BIC values provides the best fit for the data. Where these values were close between models, we selected the simplest model (i.e. the model with the fewest parameters). We restricted these comparisons to L3 growth, since the whole brains did not show significant positive growth in the L1 and L2 stages table 1.

**Table 1.** Linear regression models of larval volume, brain and mushroom body volume across the first-, second- and third-instar stages of development. *R*^2^ adj: adjusted *R*^2^. Significance codes: * *p* < 0.05, ** *p* < 0.01, *** *p* < 0.001, ‘.’ *p* < 0.1.

trait	stage	*F*-value	*p*-value	*R*^2^ adj
larval volume	L1	224.44	<2.2 × 10^−16^	0.6762
	L2	99.333	1.28 × 10^−15^	0.5514
	L3	374.65	<2.2 × 10^−16^	0.7806
brain volume	L1	6.1501	0.01472	0.04592
	L2	0.0045	0.9468	−0.0126
	L3	372.81	<2.2 × 10^−16^	0.7798
MB volume	L1	83.835	4.45 × 10^−15^	0.4364
	L2	99.097	1.35 × 10^−15^	0.5508
	L3	354.69	<2.2 × 10^−16^	0.7711

For growth in the larval body and mushroom body, we found that linear models provided the best fit to our data (table 2). This means that the growth rates in the larval body and mushroom body do not change over time in the third instar. Whole brain growth, on the other hand, was best fit with a lagged exponential model. This indicates that in the early stages of the third instar the whole brain grew very slowly. After this initial lag phase, the rate of whole brain growth increased exponentially. Taken together, these data suggest that while the larval body and mushroom body growth rates do not change with time over the third instar, the whole brain undergoes a period of little growth, followed by a second phase of rapidly increased growth in the L3.

**Table 2.** AIC and BIC for modelling larval volume, brain and mushroom body volume in the third-instar (L3) stage of development. Values for best fit are in italics. lm: linear model, poly: polynomial, exp: exponential model, explag: lagged exponential model.

trait	fit	AIC	BIC
body	*Volume.lmL3*	*20.35881*	*28.34913*
	Volume.lmL3poly	19.63951	30.29327
	Volume.expL3	30.84764	
	Volume.explagL3_100		38.83796
	Volume.powerL3	118.6141	126.6044
brain	Brain.lmL3	36.96086	44.95118
	Brain.lmL3poly	18.70613	29.35988
	Brain.expL3	19.51189	27.5022
	*Brain.explagL3_100*	*12.13046*	*22.78422*
	Brain.powerL3	156.5104	164.5007
mushroom body	*MB.lmL3*	*40.93267*	*48.92299*
	MB.lmL3poly	42.85683	53.51059
	MB.expL3	42.70775	50.69807
	MB.explagL3_100	42.80673	53.46049

### 2.2. Developmental time and growth dynamics are modulated by changes in nutrition and temperature

We next sought to determine how brain size remains robust when developmental time becomes extended as a result of altered environmental conditions. To do so, we first determined the diet and temperature conditions that produced the most differences in brain growth. We reared larvae on five different diets by diluting their total caloric content to 10%, 12.5%, 25%, 50% and a control diet containing 100%. We also used three temperatures, a lower temperature of 18°C, control temperature of 25°C or a higher temperature of 29°C. Our preliminary data showed that we could achieve the greatest range of effects by comparing the 10%, 25% and 100% diets and 25°C and 29°C rearing temperatures (electronic supplementary material, figure S3). We compared growth rates in the L3 across these six environmental conditions. Changing the diet and/or rearing temperature altered the time it took for animals to initiate metamorphosis at pupariation (white pre-pupae). Compared to animals grown under standard conditions (25°C and 100% food), animals reared on food with only 10% of the normal caloric content took the longest to pupariate (90 and 80 h after the moult at 25°C and 29°C, respectively, compared to 42 h at 25°C on 100% food). At 25°C, pupariation was delayed to 50 h after the moult when larvae were reared on 25% food. Development time was similar between the 25% and 100% food conditions at 29°C (42 h from moult to white pre-pupae).

**Figure 4.**
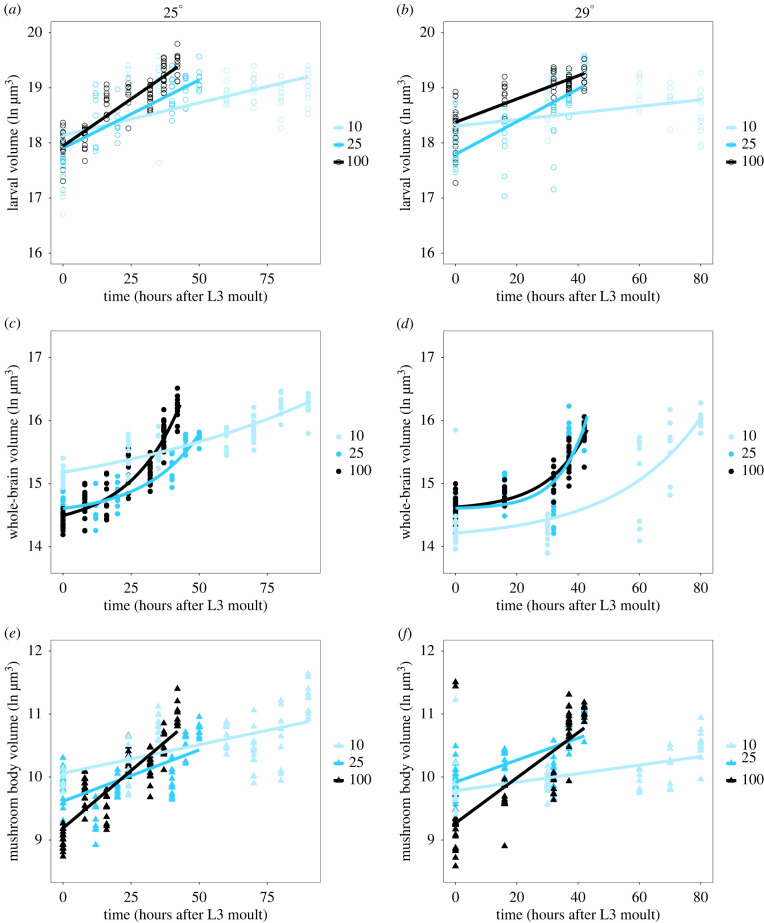
Growth rates of the larval body (*a,b*), whole brain (*c,d*) and mushroom bodies (*e,f*) over time from the moult prior to third instar through pupariation under different dietary and thermal conditions. (*a*,*c*,*e*) show three dietary conditions (10%, 25% and 100% food) at 25°C. (*b*,*d*,*f*) show the three dietary conditions (10%, 25% and 100% food) at 29°C.

Given these differences in development time across nutritional and thermal conditions, we next defined how this changed growth dynamics of the mushroom body, whole brain and larval body. For each condition, we sampled 5–7 time points across the L3 stage, with the last 2 time points corresponding to the wandering and white prepupal stages, respectively. Diluting the food reduced growth rates of the larval body at both temperatures (figure 4*a,b*, table 3). Overall, the larval body grew more slowly when larvae were reared at 29°C compared to 25°C (figure 4*a*,*b*, table 3). Larvae grew slowest on 10% food at 29°C and fastest on 100% food at 25°C (figure 4*a,b*, table 3), resulting in a significant interaction between time, food and temperature. These data provide a convenient proof-of-principle that we can alter growth dynamics by manipulating food and temperature.

**Table 3.** Growth rates of the larval body, brain and mushroom bodies depend on nutritional and thermal conditions. Larval body and mushroom body volumes were fitted with linear models (lm). Brain volumes were fitted with second-order polynomial models with time as (Time, 2, raw = TRUE); d.f. degrees of freedom; sum sq: sum of squares. Significance codes: * *p* < 0.05, ** *p* < 0.01, *** *p* < 0.001.

	sum sq	d.f.	*F*-value	*p*-value
larval volume				
time	58.358	1	402.9612	<2.2 × 10^−16^***
food	19.295	2	66.6153	< 2.2 × 10^−16^***
temp	0.051	1	0.3500	0.554400
time × food	16.805	2	58.0189	< 2.2 × 10^−16^***
time × temp	1.358	1	9.3796	0.002328**
food × temp	0.429	2	1.4828	0.228125
time × food × temp	1.755	2	6.0605	0.002532**
brain volume				
(time, 2, raw = TRUE)	89.118	2	601.0508	< 2.2 × 10^−16^***
food	2.620	2	17.6721	4.178 × 10^−8^***
temp	5.342	1	72.0532	3.331 × 10^−16^***
(time, 2, raw = TRUE) × food	13.364	4	45.0669	< 2.2 × 10^−16^***
(time, 2, raw = TRUE) × temp	6.363	2	42.9132	< 2.2 × 10^−16^***
food × temp	14.659	2	98.8697	< 2.2 × 10^−16^***
(time, 2, raw = TRUE) × food × temp	2.207	4	7.4441	8.318 × 10^−6^***
mushroom body volume				
time	51.040	1	371.8073	< 2.2 × 10^−16^***
food	2.806	2	10.2220	4.576 × 10^−5^***
temp	0.012	1	0.0880	0.7669
time × food	25.315	2	92.2044	< 2.2 × 10^−16^***
time × temp	0.081	1	0.5931	0.4416
food × temp	6.580 2	2	23.9649	1.318 × 10^−10^***
time × food × temp	0.104 2	2	0.3788	0.6849

Changing developmental time allowed us to directly test our different models. We predicted that brain structures would remain robust to changes in developmental time in one of three ways (figure 1*a*). Our first model predicted that when the developmental time was extended, brain structures would maintain their growth rates, grow to their final size and then stop growing and remain the same size until pupariation. This would be modelled best using an asymptotic regression, but could also be approximated by a negative quadratic term from a second-order polynomial regression—indicating growth rates are slowing down. In our second model, we predicted that brain structures would remain robust against changes in developmental time by altering the time at which growth is initiated, but maintaining constant growth rates. This hypothesis would be best supported by a change in the lag constant of a lagged exponential regression. Our final hypothesis proposed that brain structures would carefully tune both their rates of growth and the time they initiated exponential growth, supported by a change in both the scaling and lag constants of a lagged exponential regression or by a change in slope in a linear regression in the case of the mushroom bodies.

In the mushroom body, we found that diluting the food reduced growth rates (figure 4*e,f*; table 3), but that rearing temperature did not affect the rate of growth in this structure. This resulted in a significant decrease in growth rates for larvae grown on 10% food when compared to 25% food, as well as reduced growth rates on 25% food when compared to 100% food at both temperatures. Under all conditions, the mushroom bodies maintained linear growth trajectories. This best supports our model that at least the mushroom body compartment of the brain achieves robustness of size by carefully tuning its growth rates to adjust for changes in developmental time.

Because the whole brain showed nonlinear growth patterns, we initially modelled whole brain growth using second-order polynomials (figure 4*c,d*; table 2). Similar to the larval body and mushroom bodies, diluting the food reduced the growth rates of the whole brain with the slowest growth on 10% food for both temperatures. Rearing temperature also reduced growth rates in the whole brain (figure 4*c,d*; table 3), and the way that food affected growth rates depended on the rearing temperature. For larvae reared at 25°C, growth rates differed depending on whether they were given 25% or 100% food. At 29°C, there was no difference in growth rate between the 25% and 100% food. Thus, the whole brain shows complex responses to the combined effects of temperature and diet.

These models allowed us to further distinguish between our hypotheses. If whole brains grew to a target size and then stopped, we would expect the quadratic terms from our polynomial regressions to be negative as growth rates decreased. In all cases where the quadratic term was significant in our models, we found that the value was positive (table 4). This suggests that our first model—that brains should grow to a target size then stop—is not supported by our data.

**Table 4.** Model to test hypothesis 1 that brains maintain growth rate to target size when developmental time is extended; d.f. degrees of freedom. Significance codes: * *p* < 0.05, ** *p* < 0.01, *** *p* < 0.001. To support this hypothesis, model 1 should fit the brain/mushroom body data better than model 2 in poorer food conditions.

brain growth rate	d.f.	*R*^2^ value	*t-*value	*p-*value
food = 25, temp = 25				
Model 1	65	0.8754	−1.325	0.19
Model 2	65	0.8754	6.288	3.07 × 10^−8^***
food = 10, temp = 25				
Model 1	74	0.7718	2.425	0.0178*
Model 2	74	0.7718	1.858	0.0671
food = 25, temp = 29				
Model 1	67	0.5775	−1.312	0.194096
Model 2	67	0.5775	3.700	0.000437***
food = 10, temp = 25				
Model 1	57	0.7683	−1.939	0.0575
Model 2	57	0.7683	5.447	1.13 × 10^−6^***

We can distinguish between our second and third models using the lagged exponential growth models using the formula $\ln(\mathrm{whole}\;\mathrm{brain})=\;a+\;e^{\left(\mathrm{Time}-b/c\right)}$, where *a* is the intercept, *b* is the lag constant and *c* is the scaling constant. If brains remain robust to changes in developmental time by altering the time at which they turn on growth (hypothesis 2, figure 1), we would expect the lag constant *b* to change, but not the scaling constant *c*. Hypothesis 3 would be supported if both the lag constant *b* and scaling constant *c* changed with altered developmental time (figure 1).

We fit our whole brain growth data with lagged exponential curves and explored whether the lag and scaling constants differed across our six environmental conditions (table 2). We then conducted pairwise comparisons between whole brain growth curves either at the same temperature but across different diets, or on the same diet but across the two temperatures. We asked whether fitting specific lag and scaling constants for the curves for each condition improved the fit to the data. For the comparisons between the 10% food and either the 25% or the 100% food, the lag constants were too dissimilar to find a common coefficient, resulting in a failure to resolve a null model. While this suggests that the lag constants differ in these comparisons, we cannot formally test for this. However, both the lag constants (1 instance) and the scaling constants (5 instances) differed significantly between conditions for whole brain growth (table 5). Taken together, our data best support a model where both the timing at which exponential growth begins and the growth rate are carefully tuned to adjust for differences in developmental time.

**Table 5.** Model to test that brains remain robust to changes in developmental time by changing the time at which they turn on growth (hypothesis 2) or by changing both growth rates and the time at which they turned on growth changed (hypothesis 3). Significance codes: * *p* < 0.05, ** *p* < 0.01, *** *p* < 0.001. To support hypothesis 2, the lag constant (b) should change, but not the scaling constant (c) and hypothesis 3, if both (b) and (c) changes with altered developmental time. We applied Holm's adjustment to the *p*-values to account for multiple tests.

comparison		any constant differs	lag or scaling constant differ	lag constant differs	scaling constant differs	intercept differs
temperature (°C)	food (%)	*F-*value (all constants the same)	(a varies)	(a and c varies)	(a and b varies)	(b and c varies)
25	25 & 100	43.315***	48.136***	4.6947	0.0854	—
25	10 & 25	does not resolve	does not resolve	does not resolve	2.772***	3.4892***
25	10 & 100	does not resolve	does not resolve	does not resolve	3.673***	2.1648*
29	25 & 100	0.8435	—	—	—	—
29	10 & 25	33.158***	16.125***	does not resolve	8.7448**	4.9836
29	10 & 100	45.503***	14.132***	does not resolve	3.0147	2.8911
25 & 29	10	64.844***	29.453***	0.1876	5.2161*	—
25 & 29	25	10.522***	13.738***	1.2351	7.3676**	—
25 & 29	100	15.285***	15.828***	6.8819***	1.4653	—

### 2.3. Changing environmental conditions affects size traits in the pre-pupae

We have shown that the growth dynamics of the larval body, mushroom body and whole brain are all sensitive to environmental perturbation, but that they respond in different ways to changes in diet and temperature. We next extended these findings by examining the effects of changed environmental conditions on their final size at pupariation.

**Figure 5.**
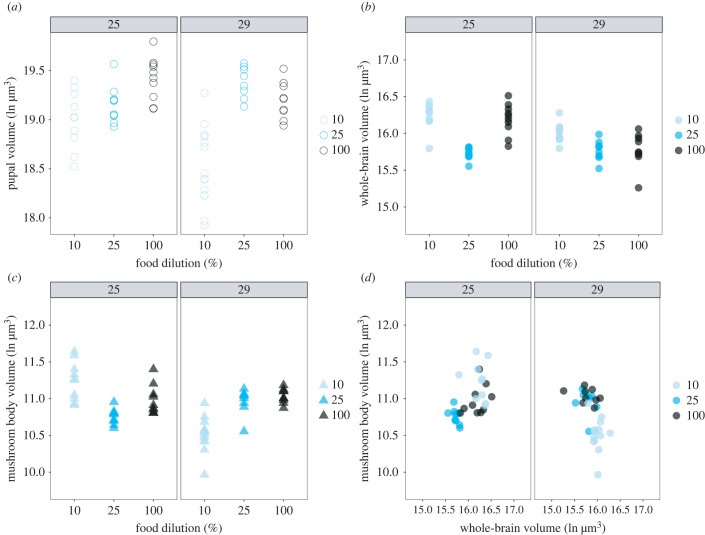
The prepupal volume of (*a*), whole brain volume (*b*) and the mushroom body volume (*c*) across nutritional (10%, 25% and 100%) and thermal conditions (25°C and 29°C). The relationship between whole brain and mushroom body volume is shown in (*d*).

Pupal body volume decreased as the food was diluted and also decreased at the higher temperature (figure 5*a*, table 6). This is what we would have expected given previously published data on the effects of diet and temperature on pupal body size [33–35]. At pupariation, we did not observe a significant effect of diet on its own for whole brain volume (figure 5*b*, table 6). However, whole brains were smaller at 29°C than at 25°C, and there was a significant temperature by diet interaction (figure 5*b*, table 6). This is due to the fact that at 25°C larval diet had no effect on brain volume whereas at 29°C, brain volume decreased with diet concentration. Mushroom body volumes at pupariation varied with diet and temperature, with increasing food concentrations and increasing temperatures negatively impacting mushroom body volume (figure 5*c*, table 6). The significant interaction between diet and temperature results from the fact that while food concentration correlates negatively with mushroom body volume at 25°C, it correlates positively with mushroom body volume at 29°C.

**Table 6.** The final relationship between whole brain and body size depends only on temperature whereas the mushroom body/body size relationship depends on both diet and temperature, with a significant two-way interaction; d.f. degrees of freedom; sum sq: sum of squares. Significant values are italicized. Significance codes: *p < 0.05, **p < 0.01, ***p < 0.001.

	sum sq	d.f.	*F*-value	*p*-value
whole brain volume				
prepupal volume	0.04509	1	0.8123	0.371511
food	0.00164	1	0.0295	0.864238
* temp*	*0.43744*	*1*	*7.8803*	0.006978**
prepupal volume × food	0.10216	1	1.8404	0.180657
prepupal volume × temp	0.02148	1	0.3870	0.536537
food × temp	0.03836	1	0.6911	0.409514
prepupal volume × food × temp	0.04492	1	0.8092	0.372417
mushroom body volume				
* prepupal volume*	*0.4282*	*1*	*7.1508*	0.009941**
food	0.1020	1	1.7032	0.197510
* temp*	*0.3106*	*1*	*5.1860*	0.026831*
prepupal volume × food	0.0162	1	0.2713	0.604640
prepupal volume × temp	0.1316	1	2.1969	0.144211
* food × temp*	*0.2992*	*1*	*4.9964*	0.029637*
prepupal volume × food × temp	0.0614	1	1.0247	0.316011
mushroom body volume by whole brain volume				
brain	0.17491	1	3.6288	0.0622218.
food	0.03948	1	0.8191	0.3695460
* temp*	*0.60644*	*1*	*12.5816*	0.0008246***
brain × food	0.04823	1	1.0006	0.3217034
* brain × temp*	*0.95303*	*1*	*19.7724*	4.495 × 10^−05^**
* food × temp*	*0.62825*	*1*	*13.0341*	0.0006789***
brain × food × temp	0.11305	1	2.3453	0.1316064

These differences in the way the whole brain and mushroom body volumes respond to diet and temperature has interesting implications for brain shape. While mushroom body volumes are remarkably robust in size on 25% and 100% foods, on 10% food they are larger for their brain size at 25°C and smaller for their whole brain size at 29°C (figure 5*d*, table 6). This highlights that brain shape changed across environmental conditions, as compartments of the brain differed in how they grew in response to these conditions.

## 3. Discussion

Individual organs vary in their response to the environmental conditions that affect adult body size [14]. Organs like the brain and genital discs are known to be nutritionally insensitive when compared to organs like the wing [10,36–39]. While we have some understanding of the genetic mechanisms underpinning robustness in size in these organs, our understanding of how these mechanisms affect the dynamics of growth was poorly understood. Further, the brain is a complex organ whose compartments do not all behave in the same manner. Functional compartments like the *Drosophila* mushroom body differ in their growth patterns as well as their nutritional plasticity from the rest of the brain. In this study, we aimed to test our predictions that the differences in proliferation between neurons of the whole brain and mushroom bodies would confer distinct growth dynamics, which could impart differences in their sensitivity to environmental conditions.

Previous studies had suggested that brain sparing occurs under stressful conditions because Jeb/Alk maintain high levels of insulin and TOR signalling in the neuroblasts [37]. These same conditions act to extend development time of the larva [40–45]. If insulin and TOR levels are at the same level in the brains of starved and fed larvae, then the brain must have additional mechanisms to prevent overgrowth when developmental time is extended. In this study, we altered development time by changing both nutrition and temperature. We proposed three hypotheses that would allow brain size to remain robust against environmental conditions. These posited that in response to changes in total development time the brain would either (i) grow to a target size and stop growing for the remainder of the growth period, (ii) delay the onset of its growth, but maintain constant growth rates even under stressful conditions or (iii) regulate both its growth rate and the time at which it switches growth on to adjust for changes in developmental time. Our data support our third hypothesis that robustness of brain size is possible because both the time at which exponential growth is initiated and the rates of growth of the brain have been altered.

Previous studies have shown that Jelly Belly (Jeb)/ Anaplastic Lymphoma Kinase (Alk) signalling acts to induce growth in the brain under starvation conditions by activating the insulin signalling pathway downstream of the insulin receptor [37]. By keeping insulin signalling on under poor nutrition, Jeb/Alk is cited as being responsible for brain sparing in *Drosophila.* Our results imply that Jeb/Alk signalling, which is responsible for brain sparing in *Drosophila*, plays a more nuanced role than previously described. Rather than simply maintaining high levels of insulin signalling, Alk signalling could be acting to adjust growth rates of the brain to match changes in developmental time. Precisely how this occurs is unknown; however, given that both insulin and ecdysone signalling are key regulators of the length of the growth period [46–50], these systemic cues could be regulating the concentration of Jeb secreted by the glial cells in accordance with the degree to which development is delayed. Other organs that show robustness in final size could be responding to environmental conditions in a similar fashion. For example, we would predict the genital discs maintain robust final size by tuning their growth rates to account for extended growth periods under poor nutrition or thermal stress.

While the size of the pupal brain is robust against environmental conditions, this does not mean that brain growth is insensitive to environmental stress. Nutritional signals are important for neuroblasts to exit quiescence and re-initiate proliferation in the larval stages [19,36,51]. Cues from the fat body, the insect equivalent of the adipose tissue and liver, signal to glial cells in the brain, which in turn produce insulin-like peptides that induce the neuroblasts to recommence cell divisions [19,36,51]. Starving larvae in early instars causes most neuroblasts and glia, with the exception of the mushroom body neuroblasts, to remain quiescent [19,36,51]. This is owing to the cell-autonomous and non-autonomous growth coordination activity of PI3Kinase in the early larval stages of development [19]. After they exit quiescence, neuroblasts no longer depend on nutritional cues to maintain proliferation [31,37]. However, our data demonstrate that rates of brain growth remain sensitive to environmental cues. Whether this is due to changes in rates of neuroblast proliferation, or changes in the rates of increase in cell size within the brain is yet unclear.

Our findings demonstrate that not all compartments of the brain should be expected to respond in the same way. Comparing between whole brain and the mushroom bodies highlights how the growth dynamics of specific brain compartments can differ from the patterns observed across the brain as a whole. Some of these differences arise simply due to differences in the timing of neuroblast reactivation. Furthermore, differences in growth patterns are not unique to the mushroom body. Unlike most of the other regions of the brain, the optic lobe shows extensive plasticity in size with nutritional conditions [31,52]. This is presumably to compensate for changes in eye size across environmental conditions and is facilitated by their unique mode of development, where the optic lobe forms from neuroepithelium instead of from embryonic neuroblasts [53,54]. Proliferation of the optic lobe neuroepithelium remains sensitive to nutrition until the early third-instar transition [52], where although the total number of neurons in the optic lobe is plastic, the diversity of cell types is held constant [52] to ensure a full complement of neuronal cells types necessary for function. Given this mode of development and persistent sensitivity to nutrition, we expect that the optic lobes would also exhibit different growth dynamics from the whole brain.

Finally, the majority of studies of brain growth have focused on nutritional stress. However, several other conditions are known to extend developmental time, including temperature, oxygen limitation and larval density [7,42,55]. The mechanisms regulating extended developmental time under these conditions are less well understood, but ultimately culminate in changing the rate of ecdysone production and secretion. Previous studies have documented that reducing or eliminating ecdysone or ecdysone signalling also reduces brain size [52,56]. Thus, in addition to insulin and TOR pathways, ecdysone is likely to regulate the size of whole brains and the size of its compartments by fixing the length of their growth period and is worth investigating.

## 4. Conclusion

In this research, we sought to understand how organs achieve robust final size by exploring the growth dynamics of the brain across nutritional and thermal conditions. We found that at least one compartment of the brain can differ in its growth patterns from the rest of the brain and speculate that this might be true of other compartments. These distinct growth patterns allow specific brain regions to vary their response to changing environmental conditions. Taken together, our findings demonstrate that brain compartments achieve robustness in final size via different trajectories. Furthermore, by probing the growth dynamics of organs under environmental stress, we fill in important gaps in our knowledge of how these organs achieve robustness of final size.

## 5. Material and methods

### 5.1. Fly strains and husbandry

*Drosophila* stocks were reared at 25°C with 65% humidity, on a 12 h light/dark cycle and maintained on sucrose-yeast (SY) diet (detailed below). To drive the expression of GFP in the mushroom body neurons, we used the R21B06-GAL4 line (BDSC 68318), known to be expressed in the mushroom bodies of larval and adult brains (http://flweb.janelia.org/cgi-bin/flew.cgi; [57,58]). This line was crossed with a membrane-tagged GFP reporter (w[*]; P[y[ + t7.7] w[ + mC] = 10XUAS-IVS-myr::GFP]attP2). These stocks were obtained from the Bloomington Drosophila Stock Center, Indiana University, Bloomington.

### 5.2. Media and larval rearing and staging conditions

SY diet was prepared as reported by [59], with 100 g autolysed brewer's yeast, 50 g sucrose, 10 g agar, 1.5 ml propionic acid, 15 ml Nipagin M solution dissolved in 1 l of distilled water. In addition to the standard diet (100% SY), we exposed larvae to additional experimental diets, which contained 10% and 25% of the caloric content of the standard SY diet. These diets were made by adding appropriate amounts of the original brewer's yeast and sucrose to the same concentration of agar and water. Twenty-five per cent food contained 25 g autolysed brewer's yeast and 12.5 g sucrose, while 10% food contained 10 g autolysed brewer's yeast and 5 g sucrose, dissolved in 1 l of distilled water. All diets were allowed to cool to 60° before the preservatives (propionic acid and Nipagin M) were added and the food dispensed.

Egg collection was carried out on normal diet without additional yeast for age synchronization. One hundred to 150 eggs were transferred to a 60 × 15 mm Petri dish to control for population density. Newly hatched first instar larva were collected in 2 h cohorts starting 24 h after egg lay. These newly hatched larvae were then staged to the appropriate time before imaging for body size measurements and dissection. To collect staged L2 and L3 larvae, we collected newly moulted second- and third-larval stages, determined by their anterior spiracle morphology, in 2 h cohorts as in [49]. These larvae were then staged to the desired time before imaging and dissection. To determine the duration from third instar to the white prepupal stage, L3 larvae were observed every 8 h until all larvae pupariated. We defined pupariation as cessation of movement with evaginated spiracles. Animals were raised at a control temperature of 25°C and experimental temperature of 29°C. All experiments were performed in three replicates on a 12 h : 12 h light : dark cycle at 65% humidity.

### 5.3. Image analysis and brain size measurement

Z-stack images were obtained from brain samples using the Leica Sp8 confocal microscope, at 1024 × 1024 pixel resolution every 1 µm with a 40 x water immersion objective, numerical aperture of 1 and zoom of 1. Three-dimensional volume was reconstructed with the Imaris (Bitplane) software. Image normalization was performed to ensure standardized measurements across images with different signal intensities, and three-dimensional analysis of the brain was done by software's in-built wizard. Images were rendered, and brain size measurement was gotten as three-dimensional volumes using the surface analysis tool on Imaris.

### 5.4. Body size measurement, organ dissection and immunocytochemistry

Animals picked at the relevant time points were first placed in cold phosphate-buffered saline (PBS) solution, to immobilize them, and then imaged using a Zeiss Stemi 508 dissecting microscope before dissection. These images were analysed using the FIJI (ImageJ, v. 2.0.0-rc-69/1.52i, 2019) software. The length and width of the larva or pupa were measured using the straight-line tool, and volume was calculated using the formula *lw^2^* (length × width^2^).

After measuring each larva/pupa, we dissected out their brain in cold 1 x phosphate-buffered saline (1 x PBS) under a Leica S9E dissecting microscope according to methods previously described [60,61]. Isolated brains were fixed overnight in 4% paraformaldehyde at 4°C. After four washes in a solution of cold 0.3% Triton X-100 in PBS (PBT) for 20 min each, samples were incubated in 2% normal donkey serum block solution prior to immunostaining. The blocked tissue samples were then transferred to Acti-stain 670 Phalloidin (1 : 1200, Cytoskeleton cat. no. PHDN1) reagent diluted in PBT and normal donkey serum, and incubated on a rocking platform shaker in the dark for 2–3 days at 4°C. Prior to imaging, samples were rinsed in cold PBS, and PBS was replaced with 25% KY jelly in water solution. Samples were imaged using the Leica SP8 HyD microscope with 40 x water immersion objective.

### 5.5. Image processing and statistical analysis

Data analyses were carried out in RStudio (v. 1.2.5019 2009–2019 RStudio). We fitted our body and organ size data with both linear, using the *lm* function, and nonlinear models, using the *nls* package [62]. We used AIC and BIC to assess model fit, selecting the simplest models when these values were similar. Data visualization was conducted using the ggplot package [63] in RStudio (v. 1.2.5019 2009–2019 RStudio).

## Data Availability

Data from this paper are available as figures and tables as well as included in the electronic supplementary material [64].
